# Evidence-based public health (EBPH) health policy advising and information of the public

**DOI:** 10.25646/6505

**Published:** 2020-06-04

**Authors:** Manfred Wildner

**Affiliations:** 1 Bavarian Health and Food Safety Authority, Department of Health; 2 Ludwig Maximilian University of Munich, Pettenkofer School of Public Health

Public Health has been defined as ‘the science and art of preventing disease, prolonging life and promoting […] health […] through organized community efforts’ [1]. Reflections on the art of bringing evidence into policies and finally practice are of equal importance as theoretical endeavours in this field, which are currently pointing out the non-linearity of these processes [2, 3]. Generally, advising on the transfer of evidence in public health should consider the triangular relationship of the domains of science, practice and the political field with their differing core values [4]. This contribution hence argues for a conscious awareness of words and framing, people involved, time and timing, ideas and ideologies, settings and context and their interplay, consistent with other reports [5, 6].

Words: they limit and predispose our thinking (‘linguistic turn’) and are a frequent cause of subtle misunderstandings and ideological prejudices. People: institutions and their interests are critical, as is the ‘behavioral cohesiveness’ of actors/groups and the frequent ‘behavioural incohesiveness’ of the media [7]. Time: awareness of the space time expectations of effects in public health, ‘flipped evaluation’ of entrepreneurs, ‘reversed causality’ due to future expectations, the role of windows of opportunity [8] and the importance of short term, midterm and long term planning horizons is crucial. Ideally, evidence appraisal, policy formulation, political priorisation and adaptation, practical implementation and scientific evaluation form a logical time sequence, derived from the public health action cycle, with various entry points for policy advising. Ideas: they have great influence, e.g. as value systems in science, politics, practice or moreover as political ideologies, or in the context of a stakeholder analysis of institutions, interests, ideas and their networks [9]. Settings and context: they are important for defining the political playing field, the value system, the regional scope and administrative organizational level, the sensitivity for context influences and last not least as reference for an actors own place.

Information of the public is a necessary endeavour for at least three reasons. From a scientists’ perspective it is a responsibility resulting from public funding as well as an opportunity for advancing novel insights. From a political perspective it is an important legitimation of decision making and an effective element for obtaining a mandate for action. From a public health perspective it is ‘a fantastic opportunity to get good accurate evidence-based science into the public domain at the very moment when the wider public really cares’, contributing to effective communication and management as part of e.g. risk or crisis management [10].

The underlying vision is an interactive public health-translation network with elements such as projects, programs, partnerships, models of good practice and practice of good models, cooperative research and development, doctoral-, postdoc- and midcareer-fellowships and cooperative institution building [11].
